# Antithrombotic management in an elderly CABG patient with nephrotic syndrome: a case report

**DOI:** 10.3389/fcvm.2025.1595027

**Published:** 2025-10-24

**Authors:** Feng Xu, Liu-Cheng Li, Miao-Miao Chen, Long Zhao, Jian-Ping Zhu, Kai-Li Mao

**Affiliations:** ^1^ Department of Pharmacy, Ningbo No. 2 Hospital, Ningbo, China; ^2^ Department of Pharmacy, Sir Run Run Shaw Hospital, Zhejiang University School of Medicine, Hangzhou, China; ^3^ Department of Cardiac Surgery, Ningbo No. 2 Hospital, Ningbo, China; ^4^ Department of Pharmacy, The Quzhou Affiliated Hospital of Wenzhou Medical University, Quzhou People’s Hospital, Quzhou, China

**Keywords:** coronary artery bypass grafting, nephrotic syndrome, thromboelastography, pharmacists, pharmaceutical care

## Abstract

Coronary artery bypass grafting (CABG) is a common treatment for coronary artery disease (CAD), but it poses significant perioperative risks, including thrombosis and bleeding, especially in elderly patients with comorbidities such as nephrotic syndrome and pulmonary infection. Thromboelastography (TEG) has emerged as a valuable tool for guiding dual antiplatelet therapy (DAPT) and optimizing drug treatment strategies in these complex cases. A case presentation of a 65-year-old male patient with nephrotic syndrome and pulmonary infection undergoing CABG surgery was reported. The patient's management included TEG-guided DAPT, routine monitoring of coagulation parameters, and adjustments based on clinical findings and laboratory results. TEG effectively guided DAPT and anticoagulant therapy, ensuring appropriate coagulation status and minimizing bleeding risks. The patient's postoperative management included dual antiplatelet therapy with aspirin and clopidogrel, adjusted based on TEG results. Additional interventions included the use of proton pump inhibitors to prevent gastrointestinal bleeding and tailored antibiotic therapy for pulmonary infection. The patient's clinical outcomes improved, with stable coagulation parameters and controlled infection. Clinical pharmacists play a critical role in optimizing medication regimens and ensuring patient safety through multidisciplinary collaboration. Future studies should further explore the integration of TEG and other advanced tools in personalized pharmaceutical care for complex post-CABG cases.

## 1 Introduction

Coronary artery disease (CAD) is a prevalent condition known for its high mortality rate worldwide. Coronary artery bypass grafting (CABG) is the predominant treatment modality for CAD (1). In recent years, more than 10 million individuals in mainland China have been diagnosed with coronary artery disease, and CABG constitutes approximately 20% of all cardiac surgeries (2). CABG surgery has been increasingly performed. However, patients with CAD often have complex medical conditions, comorbidities, and increased risks after CABG surgery.

During the perioperative period, patients require vigilant monitoring for potential complications, such as thrombosis and bleeding, infection, heart failure, and liver and kidney failure. It is crucial that pharmacological therapy be monitored during this time. Pharmacists are integral members of the multidisciplinary healthcare team, collaborating closely with physicians and other providers to optimize therapeutic outcomes (3–5). To our knowledge, this is the first article to explore the rationale for using thromboelastography (TEG) and other tools in formulating drug treatment strategies for elderly patients with nephrotic syndrome complicated by pulmonary infection. It provides valuable insights and serves as a reference for clinical pharmacists involved in providing relevant pharmaceutical care.

## 2 Case presentation

### 2.1 Main symptoms

A 65-year-old male patient, with a height of 165 cm, a weight of 60 kg, and a body mass index of 22.03 kg/m^2^. The patient has suffered from nephrotic syndrome for over 10 years. He was admitted to the hospital for chest pain that had lasted for more than 4 years and had worsened over the previous week. After admission, the specific examination items for nephropathy of the patient included an anti-phospholipase A2 receptor antibody of 66.02 RU/L (negative range was <14 RU/ml) and a total 24 h urine protein of 3554.2 mg (normal range is <150 mg per 24 h). Echocardiography revealed minor regurgitation in the mitral and tricuspid valves, an enlarged left atrium, a thickened interventricular septum, a degenerated mitral valve with a small amount of regurgitation, and reduced left ventricular diastolic function (left ventricular ejection fraction of 57%) (Supplementary Figure S1A). After CABG, the left ventricular ejection fraction was 68%, with a significant improvement in the data, indicating a notable enhancement in cardiac function and left ventricular contraction (Supplementary Figure S1B). A chest x-ray revealed the following: diffuse exudative changes in the left lung, an enlarged cardiac shadow, and bilateral pleural effusion (Supplementary Figure S2). The chest x-ray fluoroscopy image of the patient on December 1, 2023, showed that the structure of both lungs was still clear, the mediastinum was centered, the lung texture was increased, and patchy high-density shadows could be seen. There were infectious lesions in both lungs, and the lower lobe of the left lung and most of the upper lobe were atelectasis (indicated by the arrow). Sputum culture and drug susceptibility (sputum) revealed Burkholderia cepacia multilocularis (++); ceftazidime: 2, levofloxacin: 1, cotrimoxazole: ≤20, minocycline: 25. The test results before and after treatment observed from the blood test are shown in Supplementary Table S1. The diagnoses at admission were coronary heart disease, acute heart failure, cerebrovascular disease, pulmonary infection, nephrotic syndrome, and gout.

### 2.2 Pharmaceutical care process

The patient was examined prior to admission. The patient's temperature was 36.8°C, heart rate was 74 beats per minute, respiration rate was 20 beats per minute, and blood pressure was 179/96 mmHg. The patient was clear-headed and had a normal jugular vein. The lips were not cyanotic, the lungs were clear, there was no dry or wet rhonchi, the anterior region of the heart was not elevated, there was no pathological murmur, there was no pericardial friction rub, and the lower limbs were moderately edematous.

**Figure 1:**
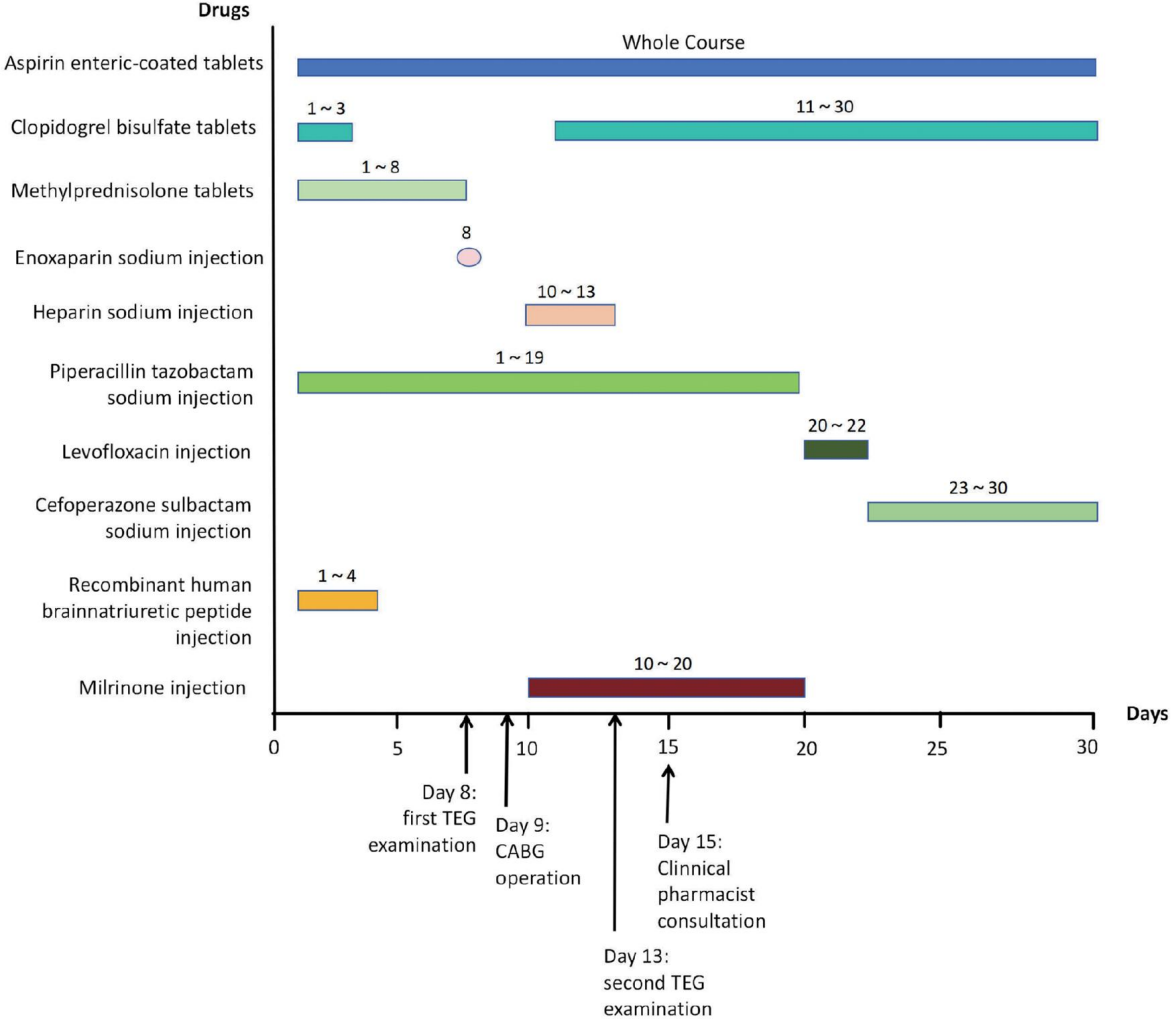
Timeline of medication treatment during hospitalization.

Routine fecal examination on the first day following admission was negative for both occult blood and red blood cells. Routine urine examination revealed the presence of occult blood (negative), protein (3+), and glucose (2+). Following joint review and discussion between the clinician and clinical pharmacist, the patient was prescribed oral aspirin (acetylsalicylic acid) enteric tablets (200 mg, once) and clopidogrel tablets (300 mg, once). On the second day, the patient was administered dual antiplatelet therapy, including enteric-coated aspirin tablets (100 mg, QD) and clopidogrel tablets (75 mg, QD) (Supplementary Table S2). Because the patient had undergone CABG surgery, aspirin was administered on day 4 as an antiplatelet agent. On day 8, the patient's coagulation function indices (PT: 12.1 s, APTT: 29.4 s) were reviewed by a clinical pharmacist, who recommended TEG to monitor the effect of the antiplatelets (Figure 1).

**Figure 2:**
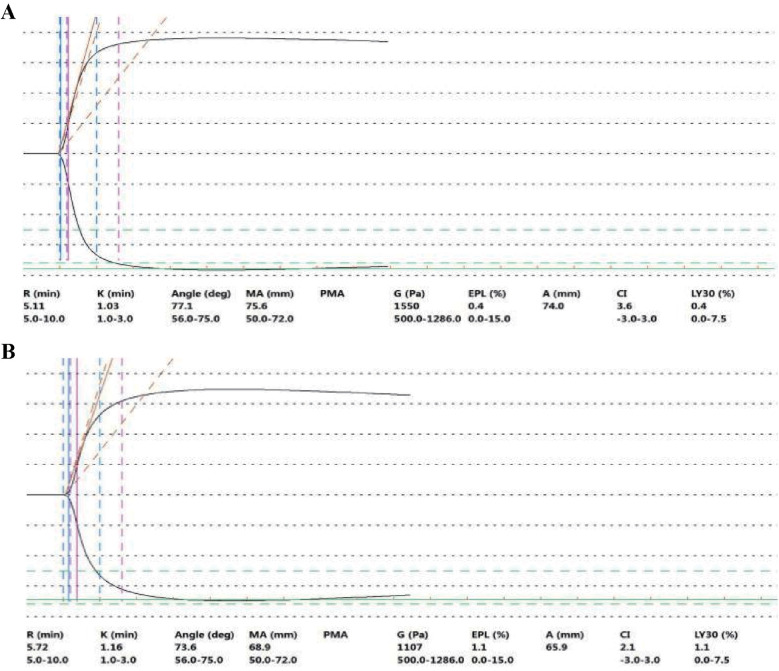
TEG plot on day 8 after admission (before surgery) **(A)** and on day 4 (after surgery) **(B)** On day 8 after admission (before surgery), the results of TEG examination (before the operation) showed that the R value was 5.11 min, the K value was 1.0 min, the Angle was 77.1°, and the MA was 75.6 mm. The activity of coagulation factors and fibrin was strong, and the platelet function was also strong. TEG examination revealed that the patient was in a hypercoagulable state. On day 4 (after surgery) (the 13th day after admission), the results of TEG examination showed that the R value was 5.72 min, the K value was 1.2 min, the ANGLE was 73.6°, and the MA was 68.9 mm. The activities of coagulation factors, fibrin, and platelet functions were all within the normal range or close to the normal values. After applying DAPT, the TEG test was within or close to the normal value (reference value) range. Reference value range: R value: 5–10 min, K value: 1–3 min, ANGLE: 53–72°, MA: 50–70 mm.

TEG revealed an R value of 5.11 min, a K value of 1.0 min, an ANGLE of 77.1°, an MA of 75.6 mm, elevated coagulation factor and fibrin activity, and robust platelet function (Figure 2A). TEG is an indicator reflecting the dynamic changes in blood coagulation and can be used to assess the risk of bleeding and thrombosis in patients. R value (normal reference range: 5–10 min): The time it takes for the first fibrin clot to form with an amplitude of 2 mm, reflecting the activity and function of coagulation factors. ANGLE (normal reference range: 53°–72°): The angle between the tangent line and the horizontal line from the formation of the blood clot to the maximum curve curvature, reflecting the function of fibrinogen. A value lower than the lower limit of the reference range indicates a decrease in fibrinogen function and a hypocoagulable state; a value higher than the upper limit of the reference range indicates the opposite. MA (normal reference range: 50 mm–70 mm): The maximum amplitude of blood clots, which assesses platelet function. A value below the lower limit of the reference range indicates decreased platelet function and a hypocoagulable state; a value above the upper limit of the reference range indicates the opposite.

Following deliberation between the clinician and the clinical pharmacist, 0.4 ml of enoxaparin sodium was injected on the same day, and DAPT combined with a short course of heparin sodium injections was administered during the postoperative period. CABG of three coronary arteries (aortas) was performed with extracorporeal circulation support on the 9th day (Figure 1). This process involves taking the great saphenous vein of the patient and establishing blood circulation channels between the aorta and the distal ends of the three diseased coronary arteries (anterior descending branch, circumflex artery, and right coronary artery), respectively, allowing blood to bypass the blocked area and supply blood to the ischemic myocardium. Heparin sodium injections of 12,500 U, qd, were administered on postoperative days 1 to 4. From postoperative day 2, enteric-coated aspirin tablets (100 mg, qd) and clopidogrel tablets (75 mg, qd) were given (Figure 1 and Supplementary Table S2). On postoperative day 4 (day 13, Figure 1), the clinical pharmacist recommended a second TEG to monitor the effect of DAPT, and the R value of the second TEG was 5.72 min, the K value was 1.2 min, the ANGLE was 73.6°, the MA was 68.9 mm, and coagulation factor activity, fibrin activity, and platelet function were within the normal range (Figure 2B). It indicates that the antiplatelet therapy is effective and not in a hypocoagulable state, without increasing the risk of bleeding. Therefore, there is no need for genotyping testing in this case for the time being. On postoperative day 6 (day 15, Figure 1), routine stool examination was 3+ for occult blood and negative for red blood cells. The clinical pharmacist was consulted and subsequently recommended that the patient take rabeprazole sodium tablets, which are oral proton pump inhibitors, to inhibit acid and protect the stomach, as gastrointestinal bleeding is a common side effect of DAPT (Figure 1). The repeat stool examination on postoperative day 8 was weakly positive for occult blood (Supplementary Table S1).

Following deliberation between the clinical pharmacist and the clinician, the patient was advised to discontinue treatment with high-dose methylprednisolone tablets, which are immunosuppressants, following cardiac surgery, and the lung infection remained uncontrolled. Consequently, bedside continuous renal replacement therapy (CRRT) was employed to maintain renal function when necessary. Considering the results of the drug sensitivity test and the patient's renal insufficiency, a cefoperazone sodium sulbactam injection was administered to manage the lung infection, while a recombinant human brain natriuretic peptide injection and a milrinone injection were administered to address heart failure, leading to adequate symptom control (Supplementary Figures S1B, S3). On December 1, 2023, the x-ray fluoroscopy image of the patient's chest showed that the structure of both lungs was still clear, the mediastinum was centered, the lung texture was increased, and patchy high-density shadows could be seen. There were infectious lesions in both lungs, and the lower lobe of the left lung and most of the upper lobe were atelectatic. The patient had a history of nephrotic syndrome. After admission, membranous nephropathy was found (with further aggravation of renal function impairment), and immunosuppressant treatment was required. However, there were infections in both lungs, leaving the patient in a dilemma between antibacterial and immunosuppressive therapy. After discussion between the clinical doctor and the clinical pharmacist, the immunosuppressant was discontinued. If necessary, CRRT was adopted to maintain renal function. The patient said he was very satisfied with the treatment process during his hospitalization and the efforts of the medical team. The patient was discharged 30 days later.

## 3 Discussion

Following CABG surgery, a comprehensive evaluation of thrombosis and bleeding risk is imperative. This evaluation is based on the patient's specific condition and considers pertinent risk factors. A rational antithrombotic treatment plan is also typically necessary. The patient, who was an elderly male with CAD in conjunction with nephrotic syndrome and Burkholderia cepacia pulmonary infection, was scheduled to undergo CABG surgery. In terms of ischemic risk, the patient's main risk factors were advanced age, a history of previous myocardial infarction, and acute heart failure. In terms of the risk of hemorrhagic transformation, the patients' main risk factors were advanced age, antiplatelet therapy, nephrotic syndrome, and dialysis treatment. The patient was at high risk for both embolism and bleeding, and the antithrombotic regimen had to be carefully designed. After CABG surgery, antiplatelet therapy was required because they were crucial in preventing transplanted blood vessels-related thrombotic events.-related thrombotic events. Furthermore, these therapies have been shown to increase transplanted blood vessels patency rates and promote clinical regression in patients.

In terms of the selection of antiplatelet drugs, previous studies revealed that dual antiplatelet therapy involving aspirin in combination with platelet P_2_Y12 receptor antagonists exerts a synergistic effect. This effect not only helps to overcome aspirin resistance but also prevents thrombosis in bridge vessels, craniofacial vessels, and peripheral vessels (6). According to the 2021 ACC/AHA/SCAI Guideline for Coronary Artery Revascularization, DAPT with aspirin plus clopidogrel remains the default after isolated CABG regardless of age, but the bleeding-risk–adjusted duration is strongly recommended in elderly or frail patients (7). However, the efficacy of aspirin in combination with clopidogrel or aspirin in combination with ticagrelor remains a subject of debate (8, 9). In patients exhibiting high ischemic risk factors, platelet function-guided DAPT step-up therapy can be employed, whereas platelet function-guided DAPT step-down therapy can be utilized in patients at high risk of bleeding (10). Zhao Shuwu (11) and others reported that thromboelastography facilitates the rational administration of antiplatelet drugs, thereby effectively reducing the incidence of ischemic events. Furthermore, Tuman, K.J (12). reported that TEG can provide more comprehensive information on postoperative coagulation and fibrinolysis function, accurately reflect the patient's coagulation function and the imbalance of coagulation function *in vivo*, and is a practical and effective monitoring tool that can guide the timely treatment of patients at high risk of bleeding.

In this case, following the patient's admission to the hospital, his oxygenation status, ischemic risk factors, high bleeding risk, and long-term side effects of Ticargrelor, such as dyspnea, were considered. Thus, DAPT therapy with aspirin and clopidogrel was initiated, as his stool was negative for occult blood. Clopidogrel was discontinued for five days prior to the CABG procedure, and aspirin was maintained as a means of antiplatelet therapy. After surgery, dual antiplatelet therapy consisting of aspirin and clopidogrel was resumed on day two, and routine stool and urine tests revealed no occult blood. This therapeutic strategy is consistent with that described in previous studies by the American College of Chest Physicians (13), and the patient's perioperative antiplatelet medication was appropriate. An examination of the thrombodynamic data revealed that the patient's coagulation factor activity, fibrin activity, and platelet function were strong in response to antiplatelet therapy, indicating the presence of thrombotic risk factors. Given the patient's elevated uric acid levels, high blood creatinine, and other pertinent factors, the administration of ticagrelor is not applicable, as ticagrelor can interfere with uric acid metabolism, leading to elevated blood uric acid levels; moreover, its active metabolites rely on kidney clearance, and drug clearance slows down and the risk of bleeding increases in patients with this kidney disease. Consequently, a short course of anticoagulants was recommended as an alternative to DAPT. With respect to the selection of anticoagulants, the American Heart Association asserts that warfarin should not be used as a standard of care following CABG to increase bridge vessel patency unless the patient exhibits a combination of indications for prolonged anticoagulant therapy, including atrial fibrillation, thrombosis of the systemic or pulmonary circulation, and prior prosthetic valve surgery (14).

Sembi N (15). et al. reported that although oral warfarin or rivaroxaban combined with aspirin for anticoagulation did not reduce the incidence of transplanted blood vessels, it did, however, reduce the risk of MACEs. Parker et al. systematically reviewed and pointed out that the prophylactic use of LMWH is the highest-rated evidence-based regimen in the prevention of thromboembolism in nephrotic syndrome combined with hypercoagulable state (16). In the present case, the patient had concomitant cerebrovascular disease and renal insufficiency (creatinine clearance 15.99 ml/min to 28.66 ml/min), non-vitamin K antagonist oral anticoagulants (NOACs) are not recommended. The patient was administered a short course of anticoagulants, including intravenous unfractionated heparin, in conjunction with dual antiplatelet therapy comprising aspirin and clopidogrel during the postoperative period. This treatment approach aligns with expert consensus (17) and the findings of the study by Dimitriadis, S. et al. (15, 18, 19), The most prevalent bleeding complication associated with DAPT is upper gastrointestinal bleeding, and the utilization of proton pump inhibitors (PPIs) has been proven to reduce the risk of gastrointestinal injury in patients and prevent bleeding and recurrent bleeding (20). Some PPIs competitively inhibit the antiplatelet effect of clopidogrel via CYP2C19, which may affect its clinical efficacy. When combined with clopidogrel, PPIs that are less affected by CYP2C19, such as rabeprazole and pantoprazole, are recommended (10). The patient presented with symptoms of upper gastrointestinal bleeding on the sixth postoperative day. In light of the impact of PPIs in combination with clopidogrel, rabeprazole sodium was administered to suppress acidity and safeguard the stomach. The patient's perioperative antithrombotic medication regimen was generally appropriate.

Shih (21) and Lin Lizhu (22, 23) demonstrated that CABG surgery is associated with a perioperative lung infection rate ranging from 3.10% to 5.78%. Amini S (24) and Yang Limeng (25) further reported that acute kidney injury (AKI) constitutes one of the most prevalent complications following CABG, with advanced age and the utilization of extracorporeal circulation identified as risk factors. The patient in question had coronary artery disease with nephrotic syndrome (membranous nephropathy) and Burkholderia cepacia multilocularis infection in the lungs. The patient underwent CABG with extracorporeal circulation support, a procedure associated with high surgical risks and difficult postoperative management. The treatment of nephrotic syndrome (membranous nephropathy) frequently necessitates the utilization of immunosuppressive medications. In cases where the immune system is suppressed, this can have a deleterious effect on the body's ability to combat infection in the lungs. Provided that the patient provided informed consent, a risky treatment strategy was selected on the basis of the patient's specific condition and willingness to undergo surgery. First, immunosuppressive treatment (methylprednisolone) was stopped before and then resumed after CABG, considering that the patient's pulmonary infection was not yet controlled. Then, symptomatic supportive treatments, such as anti-infectives, anti-congestive heart failure medications, strong diuretics, and other therapeutic treatments, were given in combination with antithrombotic therapy and hemodialysis. Following treatment, except for creatinine clearance, routine blood test results, cardiac function, coagulation function, and other indices improved. Consequently, the patient was discharged from the department. The patient was then managed in the nephrology department and discharged with improved creatinine clearance. The overall management strategy following CABG was deemed to be more satisfactory.

Although this case provided the participation of clinical pharmacists in the individualized antithrombotic management of CABG patients, there are still some limitations. Firstly, this study is a single-case design and lacks comparisons with control groups or cohorts. Second, the lack of long-term follow-up data makes it impossible to understand the long-term safety and effectiveness of the DAPT strategy. Thirdly, the patient's combined symptoms and drugs are complex, making it impossible to determine the true independent benefits of TEG-guided DAPT. Fourth, genotypes such as CYP2C19 and ABCB1 were not detected, and the clopidogrel metabolic type could not be ruled out.

In the future, incorporating genotype-guided strategies into future DAPT research holds promise for advancing precision medicine in postoperative antiplatelet care following CABG. Additionally, alternative strategies can further enhance the efficacy of dual antiplatelet therapy; for instance, thromboelastography platelet mapping was demonstrated as a valuable preoperative tool to reduce transfusion requirements by optimizing the timing of CABG surgery in patients on dual antiplatelet medication (26, 27). Prospective, multicenter, randomized controlled trials need to be conducted in the future to include high-risk CABG populations such as those with kidney disease, infection, and advanced age, and to compare TEG (or TEG-PM) guidance *vs.* the impact of the standard protocol on the thromb-bleeding composite endpoint, blood transfusion requirements, and length of hospital stay to provide high-level evidence-based evidence. Currently, neither Chinese nor European guidelines explicitly recommend a standardized method for monitoring DAPT or delineate the clinical utility of platelet-function assays in guiding antithrombotic efficacy. In this case, TEG was employed as an ancillary tool to inform DAPT decisions—an approach that merits exploration but remains strictly adjunctive rather than definitive. Large-scale, population-specific studies are still required to clarify its clinical value.

## 4 Conclusion

This study underscores the helpful role of TEG in antithrombotic therapy for elderly patients with nephrotic syndrome following CABG. The successful clinical outcomes observed in this case highlight the importance of a multidisciplinary approach, with clinical pharmacists serving as key collaborators in optimizing medication strategies and ensuring patient safety. However, the role of role of TEG remains adjunctive and not definitive in guiding DAPT. Future research should focus on validating the utility of TEG in larger cohorts and exploring its potential in combination with other advanced diagnostic tools to tailor personalized pharmaceutical care.

## Data Availability

The original contributions presented in the study are included in the article/Supplementary Material, further inquiries can be directed to the corresponding author/s.

## Supplementary material

Supplementary Figure S1 Transesophageal echocardiography examination on day 1 (before surgery) (A) and on day 23 (after surgery) (B).

Supplementary Figure S2 Chest X-ray examination on the 9th day after admission.

Supplementary Figure S3 Chest CT examination on day 23 of hospitalization.
