# Supplementary figures and images for: Antithrombotic management in an elderly CABG patient with nephrotic syndrome: a case report
Source: Front Cardiovasc Med. 2025 Oct 24;12:1595027. doi: 10.3389/fcvm.2025.1595027 (PMC12592082; doi:10.3389/fcvm.2025.1595027)

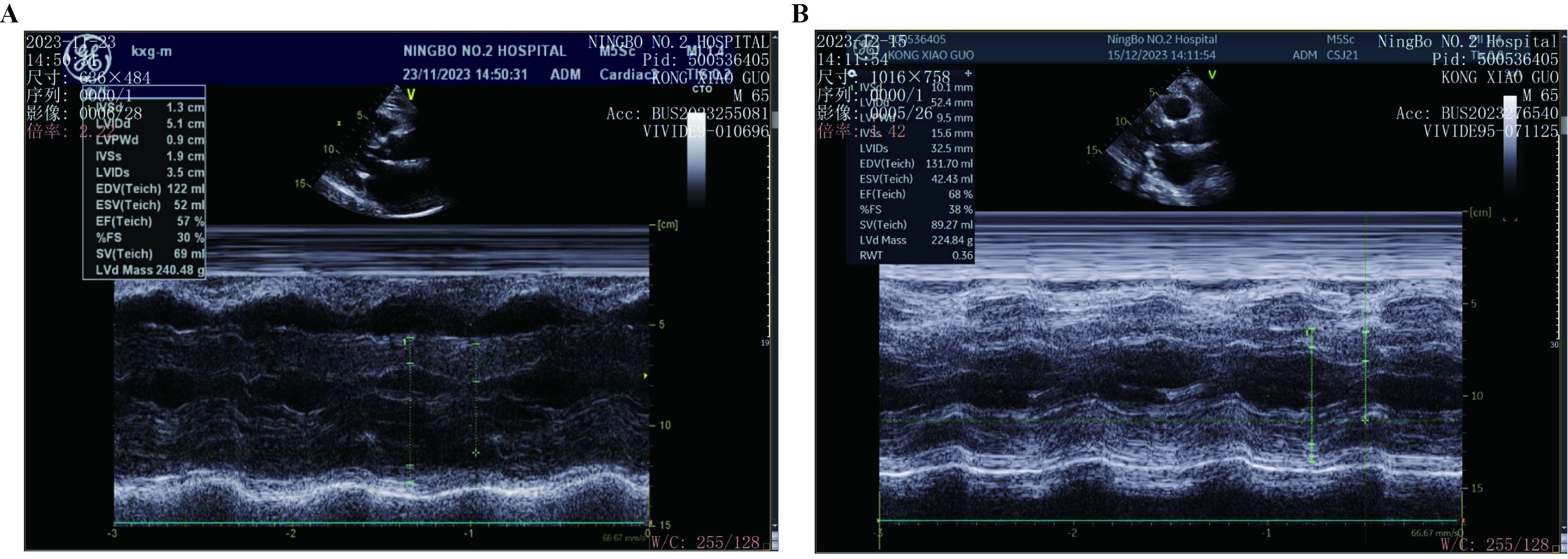

Supplement: Supplementary Figure S1 — Transesophageal echocardiography examination on day 1 (before surgery) (A) and on day 23 (after surgery) (B). [file Image1.tif]

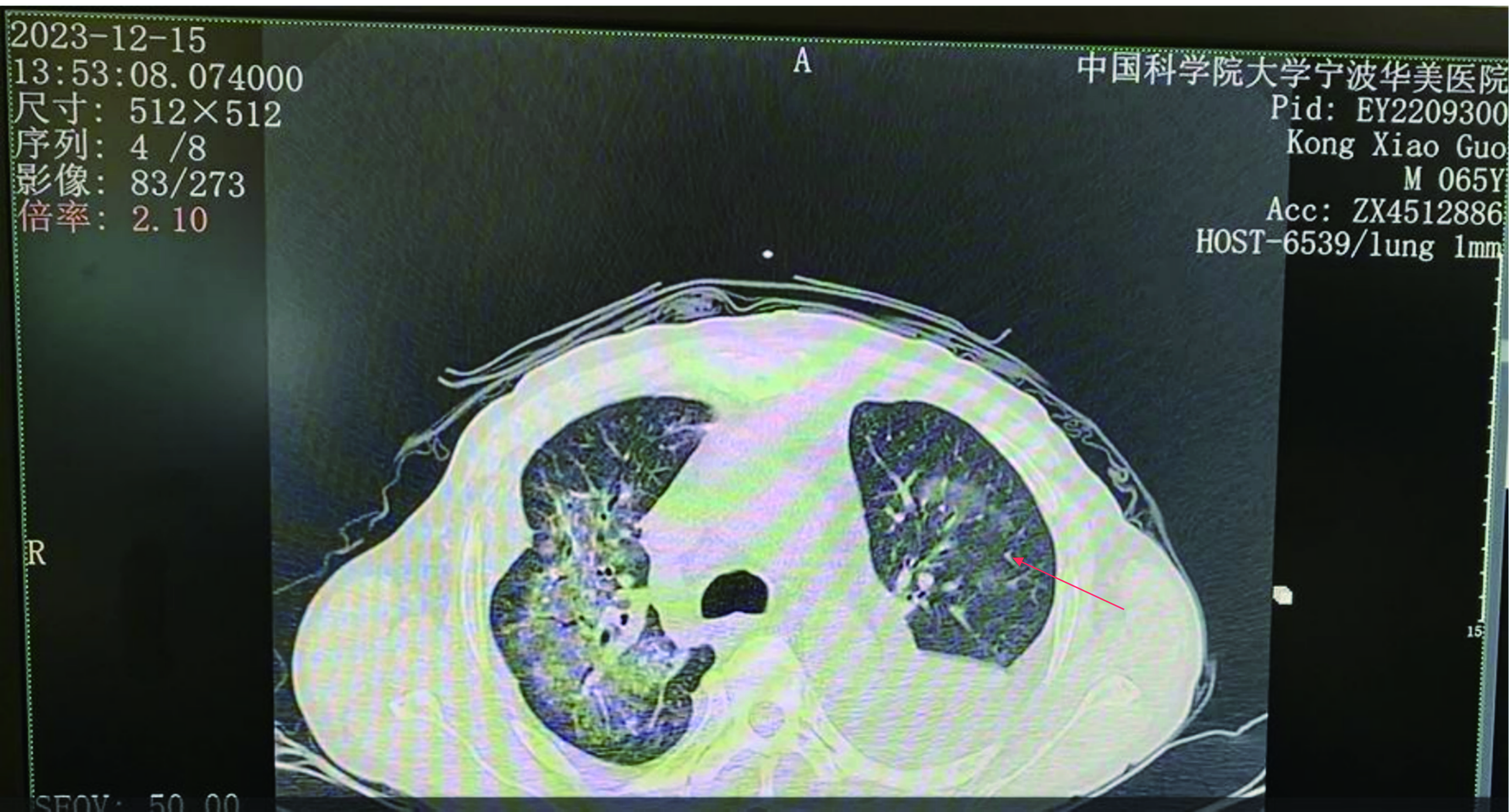

Supplement: Supplementary Figure S3 — Chest CT examination on day 23 of hospitalization. [file Image3.jpeg]
