# Supplementary material for: Antithrombotic management in an elderly CABG patient with nephrotic syndrome: a case report
Source: Front Cardiovasc Med. 2025 Oct 24;12:1595027. doi: 10.3389/fcvm.2025.1595027 (PMC12592082; doi:10.3389/fcvm.2025.1595027)
Supplement: Supplementary file 4 [file Table1.docx]

**Supplementary Table 1. Test indicator comparison.**

| **Test and inspection indicators (normal reference value)** | **Pre-treatment value** | **Post-treatment value** |
| --- | --- | --- |
| White blood cell (3.5-9.5×10^9^·L^-1^) | 12.5×10^9^·L^-1^ | 6.2×10^9^·L^-1^ |
| Red blood cel (3.8-5.1×10^12^·L^-1^) | 2.83×10^12^·L^-1^ | 2.22×10^12^·L^-1^ |
| Haemoglobin (115-150 g·L^-1^) | 88 g·L^-1^ | 66 g·L^-1^ |
| Platelet (125-350×10^11^·L^-1^) | 79×10^11^·L^-1^ | 224×10^11^·L^-1^ |
| Aspartate aminotransferase (13-35 U·L^-1^) | 20 U·L^-1^ | 34 U·L^-1^ |
| Alanine transaminase (7 -40 U·L^-1^) | 17 U·L^-1^ | 15 U·L^-1^ |
| Albumin (40.0-55.0 g·L^-1^) | 17.7 g·L^-1^ | 19.8 g·L^-1^ |
| Creatinine clearance (≥90 ml·min^-1^) | 24.97 ml·min^-1^ | 15.99 ml·min^-1^ |
| High-sensitivity troponin (＜0.0175 ng·ml^-1^) | 0.5414 ng·ml^-1^ | 0.0370 ng·ml^-1^ |
| B-type natriuretic peptide (＜100 pg·mL^-1^) | 2953 pg·mL^-1^ | 872 pg·mL^-1^ |
| Stool routine (—) | — | — |
| Urine occult blood (—) | — | — |
| Urine protein (—) | +++ | ++ |
| Urine glucose (—) | ++ | — |
