# Supplementary material for: Antithrombotic management in an elderly CABG patient with nephrotic syndrome: a case report
Source: Front Cardiovasc Med. 2025 Oct 24;12:1595027. doi: 10.3389/fcvm.2025.1595027 (PMC12592082; doi:10.3389/fcvm.2025.1595027)
Supplement: Supplementary file 5 [file Table2.docx]

**Supplementary Table 2. Processes relevant to drug treatment.**

| **Drug Name** | **Norm** | **Dosage Route** | **Treatment Time** |
| --- | --- | --- | --- |
| Aspirin enteric-coated tablets | 25mg*100 pills | 200mg, st, po | D1 |
| Clopidogrel bisulfate tablets | 75mg*7 pills | 300mg, st, po | D1 |
| Aspirin enteric-coated tablets | 25mg*100 pills | 100mg, qd, po | D2-30 |
| Clopidogrel bisulfate tablets | 75mg*7 pills | 75mg, qd, po | D2-D3, D11-D30 |
| Methylprednisolone tablets | 4mg*24 pills | 4mg, qd, po | D1-D8 |
| Enoxaparin sodium injection | 0.4ml:4000AxaIU | 0.4ml, st, ih | D8 |
| Heparin sodium injection | 2ml:12500U | 2ml, qd, ih | D10-D13 |
| Piperacillin tazobactam sodium injection | 4.0g:0.5g | 4.5g, q8h, ivgtt | D1-D19 |
| Levofloxacin injection | 0.5:100ml | 0.5g, qd, ivgtt | D20-D22 |
| Cefoperazone sulbactam sodium injection | 0.5g:0.5g | 1.0g, q12h, ivgtt | D23-D30 |
| Recombinant human brain natriuretic peptide injection | 0.5mg | 1mg, qd, ivgtt | D1-D4 |
| Milrinone injection | 5mg:5ml | 10mg, q12h, iv | D10-D20 |
